# East Indian Sandalwood Oil Is a Phosphodiesterase Inhibitor: A New Therapeutic Option in the Treatment of Inflammatory Skin Disease

**DOI:** 10.3389/fphar.2018.00200

**Published:** 2018-03-09

**Authors:** Manju Sharma, Corey Levenson, John C. Browning, Emily M. Becker, Ian Clements, Paul Castella, Michael E. Cox

**Affiliations:** ^1^The Vancouver Prostate Centre, Vancouver Coastal Health Research Institute, Vancouver, BC, Canada; ^2^Santalis Pharmaceuticals, Inc., San Antonio, TX, United States; ^3^Texas Dermatology and Laser Specialists, San Antonio, TX, United States; ^4^Department of Urologic Sciences, The University of British Columbia, Vancouver, BC, Canada

**Keywords:** psoriasis, atopic dermatitis, eczema, cancer, phosphodiesterase, skin organoid, anti-inflammatory, anti-proliferative

## Abstract

Cyclic adenosine monophosphate phosphodiesterases (PDEs) regulate pro-inflammatory cytokine production. One isoform, PDE4, is overactive in chronic relapsing inflammatory skin diseases: psoriasis and eczema/atopic dermatitis, and in several cancers. East Indian sandalwood oil (EISO) has significant anti-inflammatory properties. Here, we report that 75% of pediatric eczema/atopic dermatitis patients treated with topical EISO formulations achieved a >50% reduction in their Eczema Area and Severity Index score. EISO treatment of a psoriasis model reduced PDE4 expression and reversed histopathology. EISO directly inhibited PDE enzymatic activity *in vitro*. In lipopolysaccharide-stimulated human dermal fibroblast, BEAS-2B, A549, and THP-1 cells, EISO suppressed total cellular PDE activity, PDE4, and 7 transcript levels, nuclear factor kappa B (NF-κB) activation, and pro-inflammatory cytokines/chemokine production. These results suggest that EISO anti-inflammatory activity is mediated through suppressing PDE activity, thus facilitating cAMP-regulated inhibition of NF-κB and indicate EISO as an attractive natural therapeutic for chronic and acute inflammatory disorders.

## Introduction

Psoriasis (PS) and atopic dermatitis (AD) are chronic or recurrent inflammatory dermatologic disorders that significantly impact quality of life for millions of patients and tax health care resources worldwide (Carroll et al., 2005; Mancini et al., 2008; Arkwright et al., 2013; Parisi et al., 2013). PS and AD are clearly separable disorders with AD typically presenting in early childhood and PS typically presenting in late adolescence and early adulthood (Guttman-Yassky et al., 2011; Czarnowicki et al., 2014; Noda et al., 2015; Chiricozzi et al., 2016). However, numerous environmental and genetic factors contribute to these cytokine-mediated chronic skin conditions, and it is difficult to differentiate PS and AD during the acute erythrodermic phase since both are caused by aberrant production of inflammatory cytokines/chemokines by keratinocytes and innate and adaptive immune cell populations (Schafer, 2012). Not surprisingly, therapies that target inflammatory cytokines/chemokines signaling pathways provide symptomatic benefit to PS and AD patients (Gittler et al., 2012; Lowes et al., 2014).

Existing topical and systemic medications for these disorders are not universally effective and pose adverse side effect risks requiring intermittent use and close monitoring (Hsu and Armstrong, 2014). Identification of topical anti-inflammatory agents that can abrogate symptoms would be useful for the treatment of chronic inflammatory skin disorders. Natural products remain the best source for the development of new therapies because of their structural diversity and they do not match any source of small molecules (Newman and Cragg, 2012). While widely used in aromatherapy, the essential oil from Indian sandalwood trees, *Santalum album (Santalaceae)*, commonly known as East Indian sandalwood oil (EISO), is a potent anti-inflammatory, antiseptic and astringent agent (Burdock and Carabin, 2008; Sharma et al., 2014; Bommareddy et al., 2017), properties ideal for treatment of inflammatory skin disorders as has been reported for psoriasis (Sharma et al., 2017) and acne (Moy et al., 2012).

Cyclic adenosine monophosphate (cAMP) is an important regulator of immune and inflammatory responses, and turnover of intracellular cyclic nucleotides is dependent on the activity of phosphodiesterases (PDEs) (Oldenburger et al., 2012). PDE isoenzymes levels are regulated by various stimuli including prostaglandin E2-induced expression of PDE3B, 4A4, 4A1, 4D2, and 4D3 in human T-lymphocytes (Seybold et al., 1998) and toll-like receptor 4 (TLR4)-induced expression of PDE4B2 in human monocytes (Elwood et al., 2010). Of the eleven PDE families (PSE1-11), PDE4 is the primary cAMP-specific enzyme class. It consists of four genes (PDE4A-D), each with multiple variants encoding more than twenty protein products (Wittmann and Helliwell, 2013; Blanchard et al., 2014). PDE4s are the predominant cAMP-degrading isoenzymes in hematopoietic cells (Bjorgo and Tasken, 2006). In addition to their actions in immune cells, PDE4 isoforms are modulators of proinflammatory stress responses in pulmonary epithelial cells (Seybold et al., 1998; Wright et al., 1998), vascular endothelium, keratinocytes, smooth muscle, joint chondrocytes, synoviocytes (Houslay et al., 2005; Schett et al., 2010), and particular aspects of the central nervous system (Ashrafian et al., 2010; Martinez and Gil, 2014). The therapeutic potential of PDE4 inhibition in diseases such as chronic obstructive pulmonary disease (COPD), cystic fibrosis (CF) and asthma, has long been appreciated, with PDE4B up-regulation in airway epithelial cells being implicated in COPD pathogenesis (Michalski et al., 2012).

Increased intracellular cAMP levels activate cAMP-dependent protein kinase (PKA), that in turn activate certain transcription factors, such as cAMP-response element binding protein (CREB), but inhibit others, such as nuclear factor kappa B (NF-κB) (MacKenzie and Houslay, 2000). PKA does not appear to inhibit NF-κB-driven transcription by disrupting IkBα degradation or NF-κB DNA binding activity (Takahashi et al., 2002). Rather, NF-κB transcriptional activity may be inhibited either directly, through phosphorylation of the NF-κB subunit, p65, on serine 536 of the C-terminal transactivation domain (p-p65) (Takahashi et al., 2002) and blocking p65 nuclear translocation (Abdollahi et al., 2011), or indirectly, through phosphorylation and competition for the transcriptional coactivator CREB binding protein (CBP), or its homologue, p300 (Parry and Mackman, 1997).

Phosphodiesterases are upregulated and activated in PS and AD, and are implicated as key inducers of these and other inflammatory disorders (Smith et al., 2004; Schafer, 2012; Schafer et al., 2014). Aberrant expression and activation of PDE4 in keratinocytes and infiltrated leukocytes can promote PS, AD, and other conditions (Schafer et al., 2014) through immune-mediated inflammatory processes (Oger et al., 2005). While TLR activation, and production of prostaglandins can direct increased expression of PDEs, particularly PDE4 (Schafer et al., 2014), and EISO can suppress lipopolysaccharide (LPS)-mediated cyclooxygenase activity and cytokine/chemokine expression (Sharma et al., 2014), whether EISO can also block LPS-induced stress responses, such as PDE activation, is unknown. In this report we test the hypothesis that EISO may suppress these pro-inflammatory responses through inhibition of cAMP-degrading PDEs and subsequent promotion of cAMP-mediated NF-κB activation. We demonstrate that EISO can provide symptom relief for pediatric AD patients, and that EISO can suppress chronic inflammatory psoriatic skin conditions by suppressing PDE4 and 7 expression, and total PDE and NF-κB activation, in skin, blood, and lung cancer models. These results suggest that EISO may be a novel therapeutic agent for treating chronic and acute inflammatory disorders, such as PS and AD, and other inflammatory diseases.

## Materials and Methods

### Phase 2 Clinical Trials for Treatment of AD/Eczema

A single-center, open-label, proof-of-concept Phase 2 trial of a regimen of three topical EISO formulations containing 0.1% colloidal oatmeal for treatment of AD/eczema was conducted (Browning et al., 2017). The Western Institutional Review Board (WIRB) reviewed and approved the protocol and informed consent/assent documents before study start-up (WIRB Study number 20140941). All patients and/or legally authorized representatives provided written informed consent/assent, as applicable, prior to conduct of any study-related activities (WIRB Study Number is 1146975). The trial participants used a combination of bubble bath gel, soothing cream and daily cleanser for 60 days. Twenty five patients with mild, moderate or severe eczema were enrolled in the study, and 22 patients completed the treatment regimen. The patients were assessed for changes in their disease severity using changes in their Eczema Area and Severity Index (EASI) scores as described in Hanifin et al. (2001) and Leshem et al. (2015).

### Cell Culture

Primary cultures of human dermal fibroblast (DF), BEAS-2B immortalized human bronchial epithelial cells, and A549 cells (an alveolar type II epithelium-like cell line derived from a lung adenocarcinoma) were maintained in DMEM supplemented with 5% (v/v) FBS (Invitrogen, Carlsbad, CA, United States). THP-1 human acute monocytic leukemia cells were maintained in RPMI (Invitrogen)/5% (v/v) FBS. All cultures were maintained in a 37°C, 5% CO_2_ atmosphere.

### Experimental EISO Formulation

Pharmaceutical-grade EISO (lot: PISO-110904SD/SA) was obtained from Santalis Pharmaceuticals, Inc. (San Antonio, TX, United States). Constituent analysis and its compliance with the international standard for Indian sandalwood oil (ISO 3518:2002) has been previously reported (Sharma et al., 2017). EISO was diluted in dimethyl sulfoxide (DMSO) as a 10% (v/v) stock. The equivalent volumes of DMSO used as vehicle controls had no adverse effects on the cells.

### Human Full-Thickness Skin Model

Reconstituted full-thickness normal human skin and psoriatic phenotype organoid models (MatTek Corp., Ashland, MA, United States) were incubated in the manufacturer’s assay media (Grossman et al., 1989; Nograles et al., 2008) supplemented with or without EISO at 0.001 or 0.002% (v/v). Media, with or without supplementation, were changed every second day. On day four, specimens were collected for histologic and immunohistochemistry (IHC) analysis.

### Antibodies and Reagents

NF-κB p-p65 monoclonal antibody (93H1) was from Cell Signaling Tech (Danvers, MA, United States), and PDE4 polyclonal antibody (ab14628) was from Abcam (Cambridge, United Kingdom). The BIOMOL Cyclic Nucleotide PDE Assay kit was from ENZO Life Sciences, Inc. (Farmingdale, NY, United States). LPS (*Escherichia coli* 0111:B4) and 3-isobutyl-1-methylxanthine (IBMX) were from Sigma-Aldrich (St. Louis, MO, United States), and rolipram was a gift from MedChem Express (Monmouth Junction, NJ, United States).

Immunohistochemistry staining of reconstituted psoriatic and normal tissue models was conducted using deparaffinized sections subjected to antigen retrieval, followed by incubation with anti-PDE4 primary antibody (1:100). Bound antibody was detected by DAB staining using a Ventana universal secondary antibody (Ventana Medical System, Tuscan, AZ, United States). All stained slides were digitally imaged at magnification equivalent to 20×. Random fields were scored by an independent pathologist.

### cAMP PDE Activity

Total cAMP PDE activity was determined from lysates of DF, BEAS-2B, A549, and THP-1 cell at 2 and 4 h following stimulation with LPS (1 μg/ml), and EISO (0.001–0.002%), or IBMX or rolipram [10 μM; concentrations previously demonstrated to saturate for PDE inhibition (Cox et al., 1999; Oger et al., 2005)]. PDE activity was assessed using a colorimetric cyclic nucleotide phosphodiesterase assay (Enzo Life Sciences) following manufacturer’s protocol, and expressed as pmol phosphate generated per min relative to the activity of a supplied bovine brain PDE preparation standardized to total cell lysate protein content determined using the Pierce BCA protein assay kit (Thermo Fisher Scientific, San Jose, CA, United States).

*In vitro* PDE activity was assessed by modification of the Enzo Life Sciences’ cyclic nucleotide PDE assay kit. Briefly, EISO (0.001–0.002%) was added to the bovine brain PDE preparation (20 mUnits; 1 Unit = hydrolysis of 1 nM cAMP per min at 30°C, pH 7.4) and incubated in the presence of assay buffer, cAMP, and 5′-nucleotidase for 30 min at room temperature. The reaction was terminated by adding BIOMOL Green reagent and the resulting Abs. OD_620_ nm was expressed as enzymatic activity relative to a standard curve of the bovine brain PDE preparation.

### Immunofluorescence

Cells were grown on cover slips overnight, fixed in 4% (w/v) PBS-buffered paraformaldehyde, and permeabilized in 0.1% Triton X-100 phosphate buffered saline (Bio-Rad). Cells were then incubated with primary p-p65 (1:200) and visualized with Alexa Fluor 488 goat anti-rabbit IgG secondary antibody (1:1,000 Thermo Fisher Scientific; A-11008) and counterstained with rhodamine-conjugated phalloidin (1:500, Thermo Fisher Scientific; R415). Cover slips were mounted on microscopic slides using DAPI-Mounting Medium (Vector Laboratories, Inc., Burlingame, CA, United States; H-1200) and imaged as single mid-cell confocal planes using a 40× objective on an LSM 780 confocal microscope (Carl Zeiss, Canada).

### Enzyme-Linked Immunosorbent Assays (ELISAs)

Sandwich enzyme-linked immunosorbent assays (ELISAs) for TNF-α, MCP-1, IL-6, and IL-8, CXCL5 (Ray Biotech, Inc., Norcross, GA, United States), were performed on culture media from LPS stimulated DF, BEAS-2B, A549, and THP-1 cells (with and without treatment with EISO) according to manufacturer’s instructions. ELISAs for p-p65 and total p65 (RayBiotech, Inc.; PEL-NF-κB P65 S536) were performed using cell lysates from DF, BEAS-2B, A549, and THP-1 cells ± LPS/EISO under conditions indicated in the figure legend. Standard curves were constructed with supplied standards to allow conversion of Abs OD_450_ nm readings of experimental samples to pg/ml of the respective factors.

### Quantitative Real-Time PCR (qRT-PCR) for mRNA Expression of PDE Isoforms

Total RNA was isolated from DF, BEAS-2B, A549, and THP-1 cells using RNeasy kit (QIAGEN, MD, United States) according to the vendor’s protocol. cDNA was synthesized using Super Script First-Strand synthesis system for RT-PCR (Invitrogen). PDE4A, PDE4B, PDE4C, PDE4D, PDE7A, and PDE7B were amplified using real-time PCR system ABI-PRISM 7900 (Applied Biosystems, Foster City, CA, United States). Primers were designed as follows: PDE4A forward, 5′-GCTGAAGACCTCATCGTAAC-3′; reverse, 5′-ATTCTGTTTGTCCAGGAATG-3′; PDE4B forward, 5′-ATTCTGTTTGTCCAGGAATG-3′; reverse, 5′-ATGCTGGTGTAGAAAGGAGA-3′; PDE4C, forward, 5′-AGAGTGGTACCAGAGCAAGA-3′; and reverse, 5′-TGGGAGCCACCTATAACTAA-3′. PDE4D forward, 5′-CACCAAATGACCTTACCTGT-3′; reverse, 5′-AGCTCCACTGTTACCTTTCA-3′; PDE7A, 5′-GCAATATGAATTTGGCTTTC-3′; reverse, 5′-GGAAAGAGCTGCAGTCTAAA-3′; PDE7B forward, 5′-TCTTCAATACCCATGGACTC-3′; reverse, 5′-ATCCTGTGTCATTTCCTTTG-3′; 18S RNA forward, 5′-CGATGCTCTTAGCTGAGTGT-3′; reverse, 5′-GGTCCAAGAATTTCACCTCT-3′. Target gene expression was normalized to 18S mRNA levels in respective samples as an internal standard and relative transcript quantity was calculated using the ΔΔCt method of Applied Biosystems as previously described (Becker-Santos et al., 2012).

### Statistical Analysis

Phosphodiesterase activity, qRT-PCR, and ELISA assay results were compared using a one-way analysis of variance (ANOVA) followed by a Bonferroni post-test comparing only the pairs of interest if ANOVA *p*-values were significant. The post-test results are shown as ^∗^*p* < 0.05, ^∗∗^*p* < 0.01, and ^∗∗∗^*p* < 0.001 versus vehicle-treated and PDE enzyme-negative controls; ^†^*p* < 0.05, ^††^*p* < 0.01, ^†††^*p* < 0.001 versus LPS-stimulated and PDE enzyme-positive alone controls.

## Results

### Phase 2 Clinical Trial of EISO to Treat AD

**FIGURE 1:**
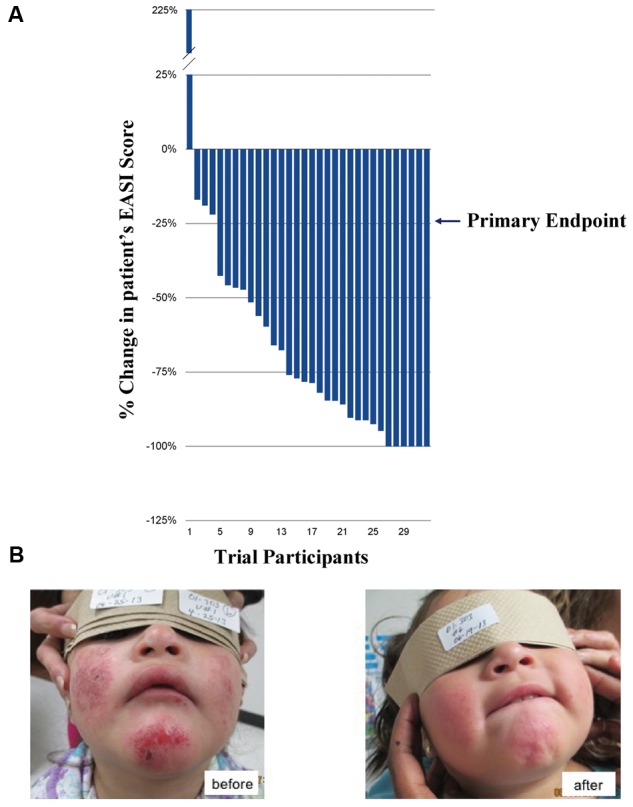
Phase 2 Clinical Trials for treatment of atopic dermatitis (AD) and psoriasis. **(A)** Percentage change in patients’ EASI scores over 8 weeks (ranked, *n* = 32). **(B)** Representative patient with severe AD (eczema) before (left) and after (right), 8 weeks of treatment with the East Indian sandalwood oil (EISO)-containing regimen. “Written informed consent was obtained from the individual for the publication of this image.”

Twenty-five pediatric patients with mild, moderate or severe AD were treated with an EISO-containing regimen over an 8-week course and assessed for changes in their disease severity using the EASI scoring method (**Figure 1A**). All but one patient (who had an allergic reaction to carpet cleaning solution prior to their final visit) showed an improvement in their condition. 87.5% of patients met the primary endpoint of the study (a 25% reduction in their EASI score) and there were no adverse events or safety issues with the treatment. The average reduction in overall scores was 67.8% (average of 6.2 EASI score reduction per patient), with 75% of patients achieving a >50% reduction in their score. 18.8% experienced a complete remission of their symptoms (a 100% reduction in EASI score). A representative patient is shown before and after treatment (**Figure 1B**).

### EISO Suppresses LPS-Induced PDE Activity

The clinical responses to EISO treatment of AD, and links between AD and PDEs described above, compelled us to examine whether EISO might affect cellular PDE activity in a panel of cell types. DF was used as established models for LPS-stimulated inflammatory responses that can be suppressed by EISO treatment (Sharma et al., 2014). THP-1 cells were used to model infiltrated leukocytes (Schafer et al., 2014). BEAS-2B cells were used to model upregulated PDE4B expression in airway epithelial cells as a contributor to the pathogenic inflammatory response of COPD (Michalski et al., 2012; Blanchard et al., 2014). A549 cells were used as a lung cancer model, since lung cancer is a frequent comorbidity in COPD and therefore share common pathogenic pathways (Adcock et al., 2011; Yang et al., 2011) including the therapeutic potential of PDE4 targeting (Pullamsetti et al., 2013).

**FIGURE 2:**
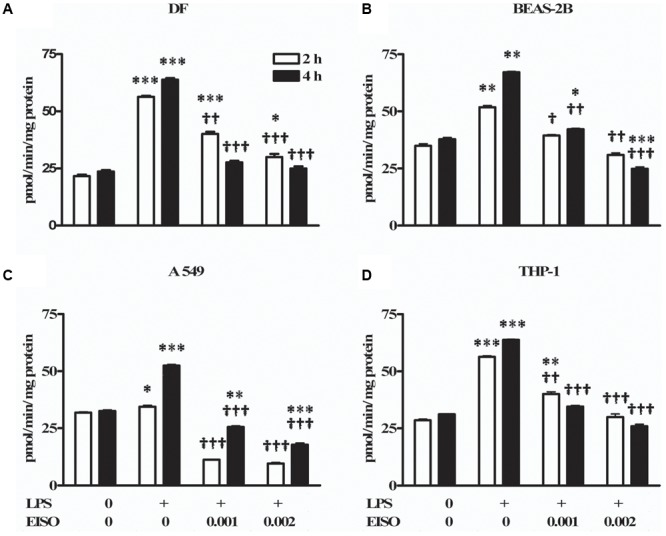
East Indian sandalwood oil suppresses lipopolysaccharide (LPS) induced phosphodiesterase (PDE) activity in cell models. **(A)** dermal fibroblast (DF), **(B)** BEAS-2B, **(C)** A549, and **(D)** THP-1 cells were treated with vehicle (0), LPS (1 μg/ml) or LPS + EISO at 0.001 or 0.002 % (v/v) for 2 h (open bars) and 4 h (filled bars). Cyclic adenosine monophosphate (cAMP) PDE activity was determined from cell lysates as described in “Materials and Methods.” All values are given as the mean ± SEM (*n* = 4). ^∗^*p* < 0.05, ^∗∗^*p* < 0.01, and ^∗∗∗^*p* < 0.001 versus unstimulated control; ^†^*p* < 0.05, ^††^*p* < 0.01, ^†††^*p* < 0.001 versus LPS-stimulated cells.

Total cAMP PDE activity was determined from cell lysates at 2 and 4 h after stimulating with LPS ± EISO (**Figure 2**). PDE activity in DF increased ∼2.5-fold after LPS exposure, while co-treatment with EISO, suppressed LPS-mediated PDE activity dose- and time-dependently, and to levels comparable to that observed in the control sample at 0.002% (**Figure 2A**). While LPS modestly increased PDE activity in BEAS-2B cells (up to 1.7-fold at 4 h), EISO suppressed LPS-mediated PDE activity at 0.001%, and to below basal levels of the control samples at 0.002% (**Figure 2B**). PDE activity in LPS-stimulated A549 cells was also modestly increased (∼1.6-fold at 4 h), and EISO treatment suppressed LPS-mediated PDE activity by at least twofold, and to levels up to a third of the basal level observed in control samples (**Figure 2C**). PDE activity in THP-1 cells increased ∼twofold after LPS exposure, and EISO treatment dose- and time-dependently suppressed LPS-mediated PDE activity to levels indistinguishable from that of the control sample at 0.002% EISO (**Figure 2D**). These observations suggest that EISO can antagonize both induced and chronic PDE activity in a spectrum of cell types.

### Pharmacological Characterization of PDE Inhibition by EISO

**FIGURE 3:**
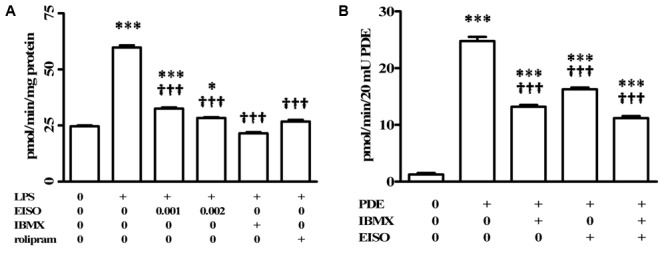
Pharmacological characterization of cAMP-PDE activity in LPS induced THP-1 cells. **(A)** THP-1 cells were treated with vehicle (0), LPS (1 μg/ml) or LPS + EISO (0.001 or 0.002%) or LPS + IBMX (10 μM) or LPS + rolipram (10 μM) for 4 h. c-AMP PDE activity was determined in the cell lysate as described in Materials and Methods. Data are mean ± SEM (*n* = 4). ^∗^*p* < 0.05, ^∗∗^*p* < 0.01, and ^∗∗∗^*p* < 0.001 versus unstimulated control; ^†^*p* < 0.05, ^††^*p* < 0.01, ^†††^*p* < 0.001 versus LPS-stimulated cells. **(B)** The ability of EISO to directly inhibit PDE was assessed *in vitro* by modification of the cyclic nucleotide phosphodiesterase assay kit as described in Materials and Methods, in vehicle control (0), bovine brain PDE (20 mU) alone, or + IBMX (10 μM), EISO (0.002%), or IBMX + EISO. Data are presented as mean ± SEM (*n* = 3). ^∗^*p* < 0.05, ^∗∗^*p* < 0.01, and ^∗∗∗^*p* < 0.001 versus no PDE control; ^†^*p* < 0.05, ^††^*p* < 0.01, ^†††^*p* < 0.001 versus PDE alone.

Phosphodiesterases are indicated as inducers of PS and AD inflammatory disorders (Smith et al., 2004; Schafer, 2012; Schafer et al., 2014), with PDE4 being specifically identified as induced in keratinocytes and infiltrated leukocytes of PS, and AD lesions (Oger et al., 2005; Schafer et al., 2014). In order to assess the contribution of PDE4 to the EISO attenuation of LPS-stimulated total cellular cAMP hydrolysis activity, suppression of total cellular cAMP PDE activity in EISO treated, LPS-stimulated THP-1 cell lysates was compared to that of cells treated with IBMX as a non-specific PDE inhibitor, or rolipram as a selective PDE4 inhibitor (**Figure 3A**). IBMX and rolipram suppressed cAMP hydrolytic activity to levels comparable to that of control and EISO-treated lysates, with the non-specific PDE inhibitor being ∼20% more efficacious.

The ability of EISO to mimic PDE inhibition by IBMX and rolipram in LPS-stimulated lysates suggested that EISO might directly inhibit PDE activity. We therefore assessed whether EISO could suppress the cAMP hydrolysis activity of bovine brain PDE (**Figure 3B**). 20 mUnits (mU) of this PDE preparation resulted in cAMP hydrolysis 25-times greater than that of buffer control. IBMX and EISO both suppressed this activity approximately 50% and the combination of EISO and IBMX only marginally further suppressed PDE activity. These results parallel the impact of EISO and IBMX on lysate cAMP hydrolysis activity seen in **Figures 2, 3A**, and suggest that EISO can efficiently antagonize potent acute PDE activation by LPS and inhibit PDE activity directly to levels comparable to that of a broad-spectrum, and a PDE4-selective, PDE inhibitor.

### Impact of EISO on LPS-Induced PDE Isoform Transcription

**FIGURE 4:**
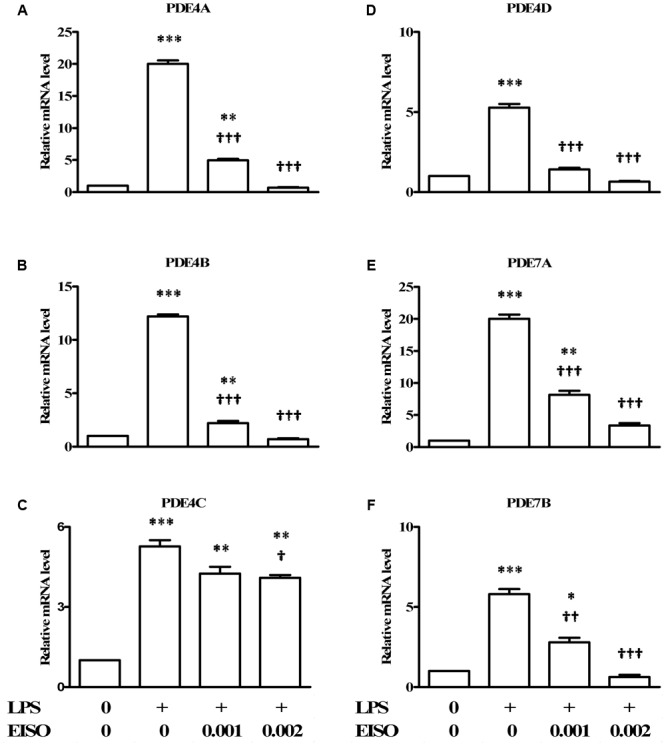
East Indian sandalwood oil suppresses expression of cAMP hydrolyzing PDE 4 isoforms. DFs were treated with vehicle control (0), LPS (1 μg/ml) or LPS + EISO (0.001 or 0.002%) for 4 h. mRNA expression of PDE isoforms: **(A)** PDE4A, **(B)** PDE4 B, **(C)** PDE4C, **(D)** PDE4D, **(E)** PDE7A, and **(F)** PDE7B, were analyzed by qRT-PCR. Relative levels of expression from representative experiments repeated thrice with similar results were normalized to 18S rRNA level. All values are given as the mean ± SEM (*n* = 4). ^∗^*p* < 0.05, ^∗∗^*p* < 0.01, and ^∗∗∗^*p* < 0.001 versus control; ^†^*p* < 0.05, ^††^*p* < 0.01, ^†††^*p* < 0.001 versus LPS-stimulated cells.

Since PDEs are known to be transcriptionally upregulated by a variety of proinflammatory stresses (Smith et al., 2004; Oger et al., 2005; Schafer et al., 2014), the ability of EISO to modulate LPS-stimulated expression of the major cAMP hydrolyzing PDEs was assessed in DF by measuring expression of PDE4A-D, 7A and 7B by qRT-PCR (**Figure 4**). LPS stimulation resulted in a twenty- to fivefold increase in pro-PDE4A-D expression, and a twenty- and sixfold increase in pro-PDE7A and B, respectively. EISO substantially inhibited expression of LPS-induced pro-PDE4A, B and D, and 7B, such that at 0.002%, transcript levels were at or below basal levels. Additionally, pro-PDE7A levels were suppressed to ∼threefold above basal levels at 0.002% EISO. Although pro-PDE4C expression was reduced ≤25% by EISO treatment, overall, these results indicate that EISO can suppress both expression and activation of the major cAMP hydrolase enzymes in response to acute LPS stimulation.

### EISO Suppresses PDE4 Expression in Reconstituted Psoriatic Human Skin Organoid

**FIGURE 5:**
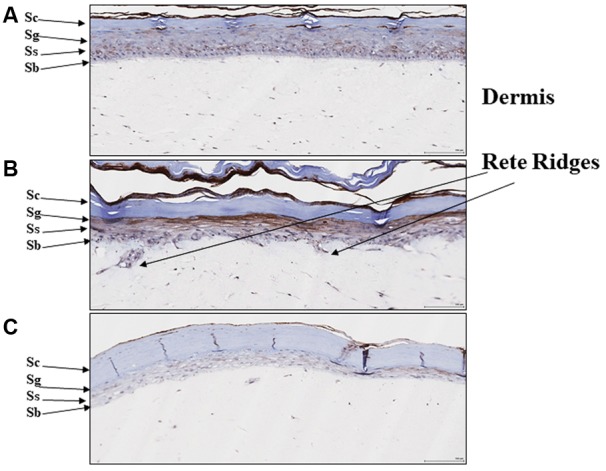
East Indian sandalwood oil alters expression and cellular distribution of PDE4. **(A)** Reconstituted normal, and **(B)** psoriatic skin after 4 days culture, or **(C)** psoriatic skin after 4 days culture + EISO (0.002%). Immunohistochemistry (IHC) of PDE4 expression in skin organoid cross sections as described in “Materials and Methods” were imaged and are shown to indicate epidermal layer: stratum corneum (Sc), stratum granulosum (Sg), stratum spinosum (Ss), and stratum basalis (Sb) with underlying dermis and Rete ridges.

We previously reported that EISO can revert the psoriatic phenotype of a reconstituted organotypic psoriatic skin model (Sharma et al., 2017). Since PDE4 expression is known to be upregulated in hyperplastic skin epidermis (Morizane and Gallo, 2012), we assessed PDE4 expression and distribution in this psoriasis model as well (**Figure 5**). After 4 days of culture, the normal skin model exhibited defined keratinocyte layers with PDE4 expression localized predominantly as light staining of stratum basalis (**Figure 5A**), while the psoriatic skin model exhibited a thickened stratum basalis layer with Rete ridges and elevated PDE4 expression in the disorganized stratum spinosum and granulosum layers (**Figure 5B**). When treated with EISO for 4 days, the psoriatic skin model exhibited well-defined epidermal layers with PDE4 expression being predominantly localized to the stratum basalis and suppressed to levels below that observed in the normal skin model (**Figure 5C**). These results indicate that the clinical observations above could be recapitulated in an *ex vivo* skin assay, and correlate restoration of normal skin phenotype by EISO to its ability to suppress PDE4 expression.

### EISO Suppresses LPS-Induced TNF-α, MCP-1, IL-6, and IL-8 Production

**FIGURE 6:**
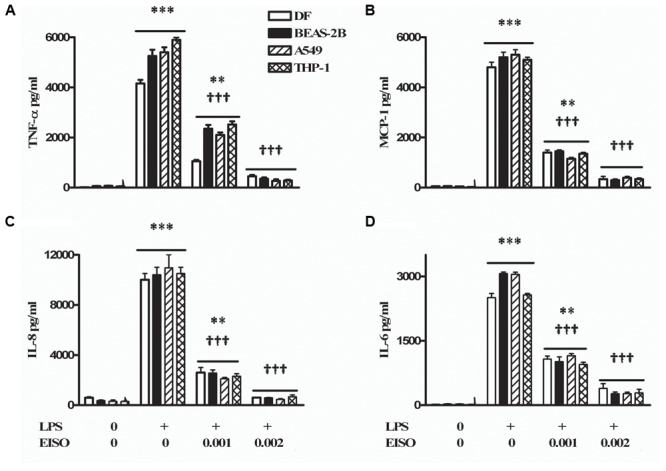
Lipopolysaccharide stimulates and EISO suppresses sentinel cytokines/chemokines production by DF (open bar), BEAS-2B (filled bar), A549 (hatched bar), and THP-1 cells (double hatched bar). Cells were treated with vehicle (0), LPS (1 μg/ml) or LPS + EISO (0.001 or 0.002%). Accumulation of TNF-α; 4 h **(A)**, MCP-1 **(B)**, IL-8 **(C)**, and IL-6 **(D)** at 24 h was determined from conditioned media by ELISA. Cytokine/chemokine accumulation was compared with levels in LPS-stimulated samples, expressed as pg/ml. Data are mean ± SEM (*n* = 4). ^∗^*p* < 0.05, ^∗∗^*p* < 0.01, and ^∗∗∗^*p* < 0.001 versus control; ^†^*p* < 0.05, ^††^*p* < 0.01, ^†††^*p* < 0.001 versus LPS-stimulated cells.

Since PDE activation is linked to proinflammatory responses, the anti-inflammatory properties of EISO were assessed on LPS-induced secretion of key cytokine/chemokine related factors (TNF-α, MCP-1, IL-6, and IL-8) in skin (DF), lung (BEAS-2B and A549), and monocytic (THP-1) cells (**Figure 6**). Using conditions previously established for induction of these cytokine/chemokines in DF and keratinocytes by LPS (Sharma et al., 2014, 2017), we observed that conditioned media contained substantially elevated levels of all four factors in these four cell lines, and that chemokine/cytokine accumulation was dose-dependently suppressed by EISO to approaching 90%. The robust suppression key cytokine-related factors production indicates that the ability of EISO to suppress proinflammatory cytokine/chemokine production is conserved across a variety of cell types.

### EISO Suppresses LPS-Stimulated NF-κB Activation

**FIGURE 7:**
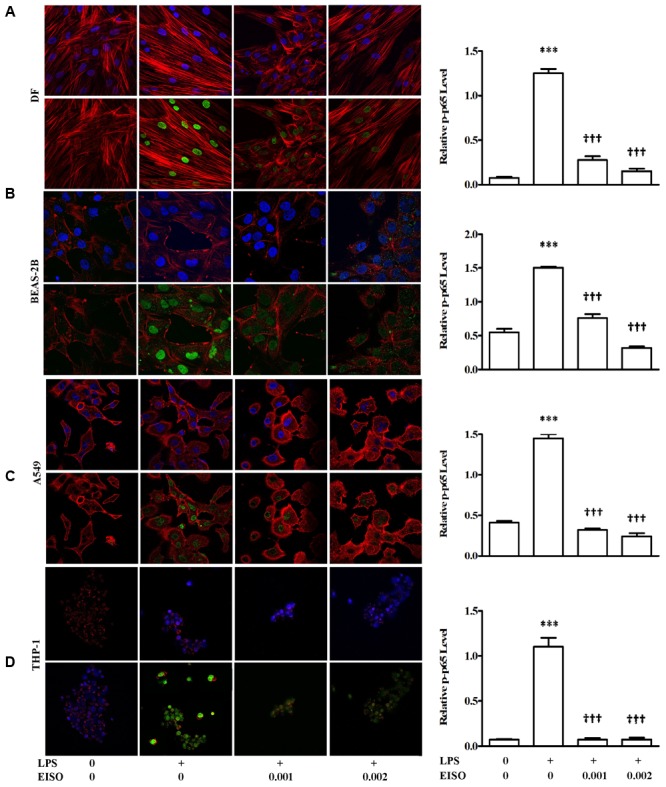
East Indian sandalwood oil suppresses p-p65 expression in DF **(A)**, BEAS-2B **(B)**, A549 **(C)**, and THP-1cells **(D)**. Cells were treated with vehicle (0), LPS (1 μg/ml), LPS + EISO (0.001 or 0.002%) for 48 h. Cells were stained for immunofluorescence imaging as described in Materials and Methods (left) to show p-p65 (green), actin filaments (red) and nuclei (blue). All stained slides were imaged using a 40× objective and identical post-capture magnification. p-p65 expression of all the four cell lines were also analyzed by ELISA (right). Data are mean ± SEM (*n* = 4).^∗^*p* < 0.05, ^∗∗^*p* < 0.01, and ^∗∗∗^*p* < 0.001 versus control; ^†^*p* < 0.05, ^††^*p* < 0.01, ^†††^*p* < 0.001 versus LPS-stimulated cells.

TNF-α, MCP-1, IL-6, and IL-8 are well characterized NF-κB transcriptional targets (Tang et al., 2017). We therefore assessed the impact of EISO treatment on NF-κB activation by measuring p-p65 levels in LPS-stimulated DF, BEAS-2B, A549, and THP-1 cells. Using an ELISA assay, all four cell lines exhibited sustained NF-κB activation after 48 h, while co-treatment with EISO suppressed LPS-mediated NF-κB activation by up to 90% in DF cells, to unstimulated levels in THP-1 cells and to levels less than or equal to that observed in control samples of BEAS-2B and A549 cells (**Figure 7**). These results were validated by immunofluorescence analysis of p-p65 expression and subcellular localization demonstrating increased nuclear p-p65 induced by LPS, and the blunting of this phenomenon by EISO at 0.001 and 0.002%. These results indicate the anti-inflammatory activity of EISO includes inhibiting activation of this central stress response modulator.

## Discussion

Erythroderma, a hallmark of inflammatory skin diseases such as PS and AD, results from immune dysregulation that leads to infiltration of inflammatory cells into the skin, interaction of cytokines, chemokines, intercellular adhesion molecules, and increased turnover of keratinocytes (Sigurdsson et al., 2000). Current treatments include emollients, vitamin D supplementation, topical calcineurin inhibitors, and topical corticosteroids (TCS) (Weidinger and Novak, 2016). Although effective, TCS may cause adverse effects, including increased susceptibility to infections and skin atrophy, and failure to respond to these topical agents can lead to patients being treated with biologics, most commonly an anti-TNF-α inhibitor. Biologics are expensive and require repeated injections; hence, the quest for less expensive alternatives is ongoing. Although the search for oral treatments has been dominated by kinase targets, small molecule PDE4 inhibitors, such as the recently approved drug, crisaborole, are generating much interest (Paller et al., 2016).

East Indian sandalwood oil has been used since antiquity as a traditional medicine, in aromatherapy, as an anti-inflammatory, astringent, antiseptic and anticancer (Dwivedi et al., 2005, 2006; Burdock and Carabin, 2008; Santha et al., 2013). Its antiseptic and anti-inflammatory properties have made it a popular traditional remedy for diverse skin problems. The anti-inflammatory benefit was shown in a phase 2 clinical study in fifty acne patients where 90% of patients showed improvement after 2 months of treatment (Moy et al., 2012). The treatment was well tolerated with a rapid reduction in reddened inflamed lesions. In addition, a similar open-label, proof of concept Phase 2 clinical trials of topical EISO formulations for treatment of AD (Browning et al., 2017) and psoriasis (Sharma et al., 2017) was found to be safe and well-tolerated and showed initial indications of efficacy. Anecdotally, the patient shown in **Figure 1** also had a concomitant MRSA infection that was resolved by the treatment. Our report here that the vast majority of pediatric AD patients met the study’s primary endpoint with no evidence of adverse events or safety issues now extends the efficacy of topical EISO to manage this dermal inflammatory pathology as well.

Although EISO’s mechanisms of action are not completely elucidated, santalols derived from EISO have been shown to have substantial anti-inflammatory properties in skin models that are linked to suppression of prostaglandin and thromboxane production and cytokine/chemokine expression (Sharma et al., 2014). PDE4 is reported to be a key modulator of pro- and anti-inflammatory mediators in both the immune (innate and adaptive) and non-immune cells (Zambon et al., 2005). LPS can stimulate an increase in total cellular PDE activity, and EISO can effectively block this PDE activation to levels comparable to that of samples treated with non-specific, and PDE4-specific inhibitors. While PDE4 and 7 are the predominant cAMP hydrolyzing classes found in immune cells, PDE4 is the dominant isoform in inflammatory cells and is highly expressed in cells of progressive psoriatic lesions (e.g., keratinocytes, vascular endothelium, and synovial membrane) (Schafer, 2012), and PDE4B is reported to be a major PDE activated by LPS stimulation (Oliva et al., 2012). Although we cannot conclude that the impact of EISO on PDE activity is an effect specific for a particular PDE subclass, our demonstration that EISO can antagonize LPS-stimulated transcript expression of PDE4 and 7 subclasses, and revert psoriatic pathology and expression of PDE4 is consistent with EISO’s anti-inflammatory properties. Our observation that ESIO could specifically attenuate cAMP hydrolysis activity in an *in vitro* enzymatic assay in **Figure 3B** suggests that the suppressed cAMP hydrolysis activity by EISO or PDE-selective inhibitors in **Figures 2, 3A** was due to an effect on the enzymes themselves. The concomitant decreased expression of transcript levels of the best annotated cAMP hydrolyzing PDE isoforms, PDE4 and 7, by EISO treatment is consistent with the known upregulation of PDE expression in response to inflammatory stresses (Smith et al., 2004; Oger et al., 2005; Schafer et al., 2014) and previous reports that EISO is a potent anti-inflammatory agent (Sharma et al., 2014, 2017; Bommareddy et al., 2017). Our demonstration of the ability of EISO to modulate protein expression and distribution of PDE4 in organoids by IHC (**Figure 5**) supports the observed EISO-mediated suppression of PDE4 transcription and indicates that EISO can suppress both activity and expression of PDE4 isoforms.

Numerous studies that describe increased PDE4 expression and cAMP hydrolysis in inflammatory syndromes. Initial studies have shown that exposure of human monocytes to LPS leads to increase in mRNA levels and increased PDE4 activity (Kotera et al., 1997; van Staveren and Markerink-van Ittersum, 2005). Similar LPS stimulation of PDE4 expression and cAMP hydrolysis activity has been demonstrated in murine PBMC and macrophages (Lugnier et al., 1999), this response was completely absent in PDE4-deficient mice (Jin and Conti, 2002). Increased PDE4 activity occurs in experimental models of systemic LPS response (Koga et al., 1995; Guo et al., 2014), and, as we have also shown here, PDE inhibitors reduce LPS-induced synthesis and release of cytokines (Dousa, 1999). The PDE4-selective inhibitor, cilomilast, is demonstrated to be effective treatments for COPD (Ouagued et al., 2005), while the first PDE4-selective inhibitor, rolipram, decreases LPS-induced lung neutrophilia in rodent models (Miotla et al., 1998; Toward and Broadley, 2001). The PDE-selective inhibitor, rolipram, is known to block ethanol-induced cAMP hydrolysis and NF-κB signaling (Gobejishvili et al., 2008). NF-κB is an essential transcriptional regulator of tumorigenesis, apoptosis, viral replication, inflammatory responses and various autoimmune diseases (Baldwin, 1996) and is part of stress responses activated by stimuli including pharmacologic agents, UV, bacterial cell wall components, growth factors and cytokines (An et al., 2002).

This first report that EISO is able to suppress LPS-stimulated NF-κB activation and resulting cytokine and chemokine expression, and to decrease transcription and activation of PDEs, suggest that EISO can antagonize proinflammatory stimuli by inhibiting the ability of PDEs to hydrolyze cAMP and thus allowing for PKA-mediated attenuation of NF-κB activation. In addition, to demonstrating the benefit of an anti-PDE activity for managing inflammatory dermal pathologies, this raises the possibility that targeting PDE4 and/or 7 could be exploited as an alternative therapeutic approach that might reduce side effects associated with targeting other PDE family members. The promising preliminary clinical trials results indicating the efficacy of EISO therapies to manage inflammatory skin disorders together with the ability of EISO to decrease NF-κB activation by suppressing proinflammatory stimulation of PDE transcription, and directly suppressing PDE enzymatic activity, provides a strong rationale for continued study of EISO as a potential new therapeutic for the treatment of inflammatory skin conditions such as PS and AD.

## Author Contributions

MC, MS, and CL were responsible for study design. MS and MC were performed all *in vitro* studies. JB and EB were conducted the clinical study. Santalis Pharmaceuticals, Inc. (CL, IC, and PC) provided EISO for the experimental use and designed the clinical study. Manuscript was written by MC, MS, and CL and all other authors provided editorial advice.

## Conflict of Interest Statement

CL, IC, and PC are employees of Santalis Pharmaceuticals, Inc. The other authors declare that the research was conducted in the absence of any commercial and financial relationships that could be construed as a potential conflict of interest.
